# Telitacicept combined with conventional therapy successfully treated MDA5-associated RPILD: A case report

**DOI:** 10.1097/MD.0000000000043293

**Published:** 2025-07-11

**Authors:** Lang Zhang, Yuhan Luo, Xingqin Shao, Rongrong Hu, Jing Yuan, Yan Zha, Xin Lin

**Affiliations:** a Zunyi Medical University, Zunyi City, Guizhou Province, China; b Department of Nephrology, Guizhou Provincial People’s Hospital, Guiyang, Guizhou Province, China; c Department of Radiology, Guizhou Provincial People’s Hospital, Guiyang, Guizhou Province, China.

**Keywords:** dermatomyositis, melanoma differentiation-associated gene 5, rapidly progressive interstitial lung disease, TACI-Fc fusion protein, telitacicept

## Abstract

**Rationale:**

Melanoma differentiation–associated gene 5 (MDA5)-associated rapidly progressive interstitial lung disease (RPILD) is a rare and life-threatening condition with no standardized treatment protocol. We report a case of successful treatment using a novel combination therapy.

**Patient concerns:**

A 69-year-old female presented with progressive dyspnea and chest tightness that had persisted for 1 year but significantly worsened over the 20 days before admission. The recent exacerbation was accompanied by coughing, expectoration, and fatigue.

**Diagnoses:**

Following a comprehensive evaluation including chest computed tomography imaging, laboratory tests, and autoantibody profiling, the patient was diagnosed with MDA5-associated RPILD.

**Interventions:**

The patient was treated with high-dose methylprednisolone pulse therapy, cyclophosphamide, and telitacicept (a novel TACI-Fc fusion protein).

**Outcomes:**

After treatment, the patient’s oxygenation index improved from 174 to 306 mm Hg. Follow-up chest computed tomography showed near-complete resolution of bilateral lung inflammation. Pulmonary function tests demonstrated improvement, with diffusing capacity for carbon monoxide reaching 63.6% of the predicted value at 6-month follow-up.

**Lessons:**

This case suggests that combination therapy including telitacicept may be effective for MDA5-associated RPILD. Further research is needed to confirm these findings and elucidate the underlying mechanisms of action.

## 1. Introduction

Idiopathic inflammatory myopathies (IIM) constitute a heterogeneous group of autoimmune disorders characterized by chronic muscle weakness, fatigue, and inflammatory infiltrates in skeletal muscles. The classification of IIM encompasses several subgroups, including dermatomyositis (DM), antisynthetase syndrome, immune-mediated necrotizing myopathy, inclusion body myositis, polymyositis, and overlap myositis.^[1]^ Among these, interstitial lung disease (ILD) associated with IIM presents a significant clinical challenge, with melanoma differentiation-associated gene 5 (MDA5)-related lung disease exhibiting particularly rapid progression.

MDA5-associated rapidly progressive interstitial lung disease (RPILD) manifests with exertional dyspnea, velcro rales, dysphagia, and digital clubbing. Pulmonary function tests typically reveal restrictive ventilatory impairment and diminished diffusing capacity.^[2]^ The radiographic presentation evolves from atypical patterns in early stages to characteristic ground-glass opacities and consolidations distributed along the pleura in advanced disease.^[3,4]^

Current management strategies for MDA5-positive DM with RPILD lack standardization. Combination therapy, comprising symptomatic support, pulse corticosteroids, calcineurin inhibitors, biologics, and in some cases, plasmapheresis, represents the primary approach.^[5]^ Rituximab, a chimeric monoclonal antibody targeting CD20-positive B cells, is frequently employed, although its use is largely extrapolated from evidence in ILD associated with other connective tissue diseases.^[6]^ Despite aggressive early intervention, treatment outcomes often remain suboptimal, underscoring the need for novel therapeutic strategies.

In this case report, we present a patient with MDA5-associated RPILD who was successfully treated with a combination therapy that included telitacicept, a novel fully human transmembrane activator and calcium modulator and cyclophilin ligand interactor-Fc (TACI-Fc) fusion protein. This approach targets both B and T cell pathways, which may offer a more comprehensive solution to the complex immunopathology of this disease.

## 2. Case

A 69-year-old female presented with progressive chest tightness and shortness of breath, which had persisted for 1 year but significantly worsened over the 20 days prior to admission. Recent exacerbation was accompanied by coughing, expectoration, and fatigue. Notably, the patient reported no generalized muscle ache or skin rash. Her medical history included well-controlled hypertension for over a decade.

Physical examination revealed wet rales in the lower regions of both lungs upon auscultation. The patient’s heart rate was 90 beats per minute, with no lower extremity edema observed.

Prior to acute presentation, baseline pulmonary function tests had demonstrated normal lung function parameters with no evidence of ILD, as confirmed by our pulmonary specialists. To exclude concurrent pulmonary infection, bronchoscopy with bronchoalveolar lavage was performed during the current admission. Bronchoalveolar lavage fluid analysis showed no evidence of bacterial, fungal, or mycobacterial infection. Cytological examination revealed inflammatory cells consistent with autoimmune lung disease without infectious organisms. Microbiological cultures and molecular testing for respiratory pathogens were negative.

**Figure 1.**
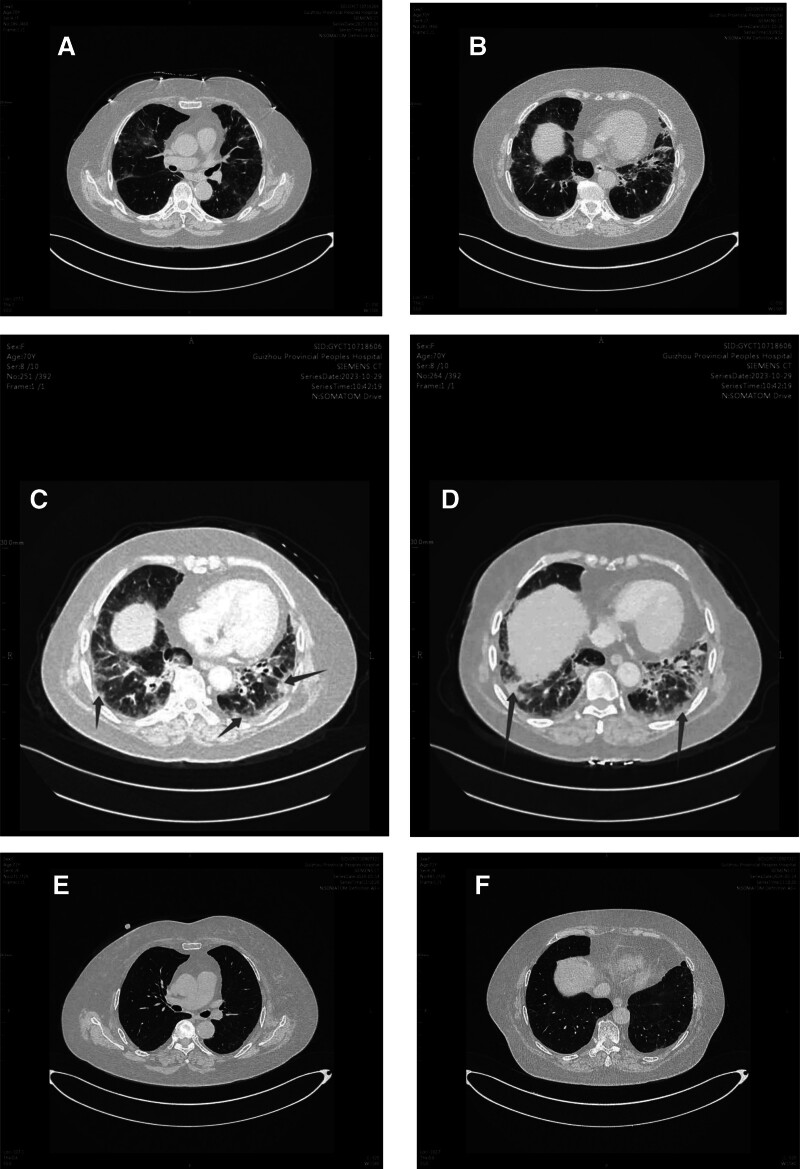
Serial chest CT images demonstrating the progression and resolution of lung inflammation during treatment. (A) Initial scan revealing multiple new inflammatory lesions in both lungs. (B) Additional view of initial scan showing extensive bilateral involvement. () Follow-up scan demonstrating multiple patchy areas of increased density in both lungs with interstitial changes, predominantly distributed subpleurally and near the pleura. (D) Corresponding view highlighting the progression of interstitial changes (arrows indicate representative lesions). (E) Final scan showing near-complete resolution of bilateral lung inflammation after treatment. (F) Corresponding view confirming significant improvement in lung condition. CT = computed tomography.

Initial chest computed tomography (CT) at presentation (Day 0) revealed diffuse ground-glass opacities in both lungs, predominantly distributed in peripheral and subpleural regions, characteristic of MDA5-positive RPILD (Fig. 1A and B). This represented a dramatic progression compared to baseline imaging performed approximately 10 months prior, which showed only limited bilateral nodular changes (Fig. 1A and B and Figure S1, Supplemental Digital Content, https://links.lww.com/MD/P389). Blood gas analysis showed an oxygenation index of 174 mm Hg (under nasal cannula oxygen therapy 2 L/min). A follow-up pulmonary artery CT on Day 3 excluded pulmonary embolism and pulmonary hypertension but demonstrated progression of the disease, with multiple patchy areas of increased density and more pronounced interstitial changes in both lungs (Fig. 1C and D).

Laboratory tests conducted on Day 6 revealed elevated antinuclear antibody titers (1:100 and 1:1000) and a positive MDA5 antibody at 1:10 in the myositis antibody profile. Serial laboratory measurements (Table 1) showed initial elevation of inflammatory markers (C-reactive protein 1.05 mg/L, interleukin-6 1.9 pg/mL) and muscle enzymes (aspartate aminotransferase 23 U/L, lactate dehydrogenase 421 U/L, creatine kinase 99 U/L).

**Table 1:** Serial laboratory measurements in a case of MDA5-associated RPILD treated with telitacicept.

Date	WBC (10⁹/L)	Hb (g/L)	N% (%)	L (10⁹/L)	CRP (mg/L)	IL-6 (pg/mL)	AST (U/L)	LDH (U/L)	CK (U/L)	a-HBDH (U/L)	Globulin (g/L)	Blood gas analysis (mm Hg)
Day 0	–	–	–	–	–	–	40	422	380	330	–	174
Day 3	–	–	–	–	3.74	–	38	397	322	300	–	186
Day 8	12.79	137	81.2	1.59	1.05	1.9	23	421	99	316	21	175
Day 13	11.9	132	81.8	1.26	0.45	1.9	40	416	151	339	22.9	–
Day 18	11.33	141	75.2	1.78	–	–	–	–	–	–	–	–
Day 21	11.84	127	88.6	0.82	–	–	–	–	–	–	19.8	306
Day 37	7.42	140	77.8	1.10	–	–	–	–	–	–	17.8	–
Day 67	10.87	134	83.5	1.11	–	–	25	540	50	456	16.3	–
Day 116	8.79	128	68.7	1.88	2.34	–	38	392	41	321	21	–
Day 142	2.16	112	33.3	1.4	0.4	–	–	–	–	–	20.7	–
Day 164	8.99	119	65.8	2.06	0.91	–	–	–	–	–	22.8	–
Day 198	11.17	116	78.7	1.71	2.12	–	–	–	–	–	24.1	–

The laboratory parameters include white blood cell count (WBC), hemoglobin (Hb), neutrophil percentage (N%), lymphocyte count (L), C-reactive protein (CRP), interleukin-6 (IL-6), aspartate aminotransferase (AST), lactate dehydrogenase (LDH), creatine kinase (CK), alpha-hydroxybutyrate dehydrogenase (a-HBDH), globulin, and blood gas analysis.

MDA5 = melanoma differentiation-associated gene 5.

**Figure 2.**
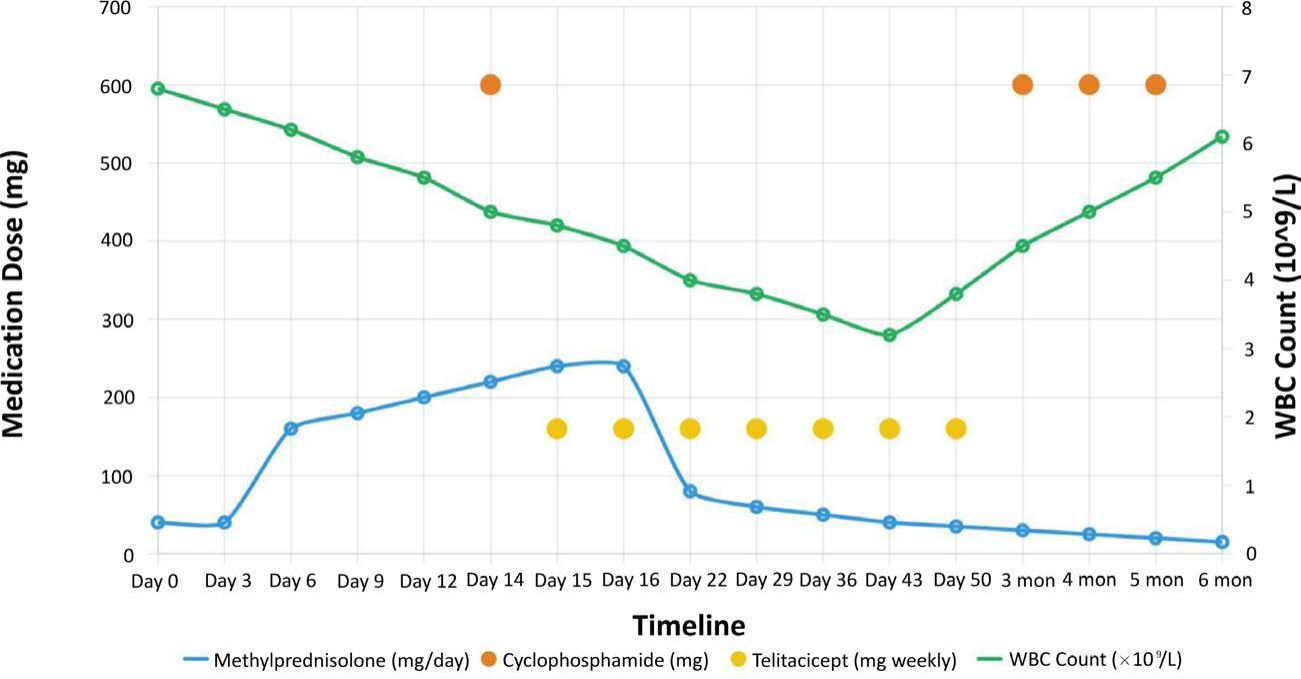
Treatment timeline and laboratory parameter changes. Treatment timeline showing daily methylprednisolone doses (blue line), cyclophosphamide administrations (orange dots: Day 14 during hospitalization, then monthly outpatient doses at 3, 4, and 5 months post-discharge), telitacicept weekly dosing (yellow dots: initiated Day 15, continued for exactly 6 weeks as per protocol), and white blood cell count changes (green line, secondary y-axis). Methylprednisolone dose was escalated from 40 mg daily to 240 mg over Days 6–14, then gradually tapered. The acute treatment phase spans Days 0–16 (Oct 31–Nov 16, 2023) with subsequent outpatient follow-up extending to 6 months.

The treatment course and laboratory parameter changes are illustrated in Figure 2. Initial treatment with methylprednisolone 40 mg daily from Day 0, oxygen therapy, and supportive care proved ineffective. Upon confirmation of MDA5-positive DM with RPILD, the treatment strategy was aggressively intensified. As shown in Figure 2, the patient received methylprednisolone pulse therapy with escalating doses (160 mg, 200 mg, and 240 mg, adjusted for body weight) from Days 6 to 18. On Day 18, intravenous cyclophosphamide (0.6 g) was administered as a single dose. This was followed by subcutaneous telitacicept (160 mg weekly) starting on Day 19. The addition of telitacicept was prompted by a significant increase in B lymphocytes observed in lymphocyte immunophenotyping.

Figure 2 also depicts the changes in white blood cell (WBC) count throughout the treatment period. Initially, the WBC count was relatively stable, but it showed notable fluctuations following the intensification of immunosuppressive therapy. A significant decrease in WBC count was observed at 4 to 6 weeks post-treatment initiation, likely associated with the potent immunosuppressive regimen. Subsequently, the WBC count gradually recovered and stabilized within the normal range by 6-month follow-up, indicating bone marrow recovery and reflecting adjustments in the immunosuppressive therapy.

Throughout the treatment course, the patient experienced no infections or injection-related adverse reactions. The patient was discharged on Day 22, with a markedly improved oxygenation index of 306 mm Hg (under nasal cannula oxygen therapy 2 L/min). Laboratory values at discharge showed improvement in inflammatory markers and muscle enzymes (Table 1).

Subsequent outpatient treatment included oral steroids, monthly cyclophosphamide (0.6 g, cumulative dose 1.8 g over 3 mo), and weekly subcutaneous telitacicept (160 mg) for 6 weeks (Days 19–61). Regular follow-up blood tests demonstrated a gradual normalization of laboratory parameters (Table 1 and Fig. 2).

Follow-up assessment at 6 months showed significant improvement. Pulmonary function tests revealed: forced vital capacity 2.36 L (104% predicted), forced expiratory volume in 1 s 2.07 L (111% predicted), forced expiratory volume in 1 s/forced vital capacity 87.72% (104% predicted), and diffusing capacity for carbon monoxide 63.6 mmol/min/kPa. High-resolution CT of the lungs demonstrated near-complete resolution of the previously observed bilateral lung inflammation (Fig. 1E and F), corroborating the clinical improvement and effectiveness of the treatment regimen. Final laboratory tests showed normal levels of inflammatory markers and muscle enzymes, with a slight increase in globulin levels (24.1 g/L) (Table 1). As illustrated in Figure 2, the WBC count had returned to normal range, indicating successful management of the immunosuppressive therapy without compromising the patient’s immune system.

## 3. Patient perspective

Throughout the treatment course, the patient reported significant improvement in her respiratory symptoms following telitacicept combination therapy. In her own words: “Prior to treatment, I experienced labored breathing even during simple daily activities such as walking short distances or climbing stairs, often requiring rest breaks. Now, I can perform moderate household activities without notable dyspnea.” The patient further noted good tolerability of the treatment regimen, with only mild facial edema during the initial high-dose methylprednisolone therapy, without other significant discomfort. Regarding the telitacicept injections, she stated: “There was occasional mild pain at the injection site, but it resolved quickly, and overall acceptability was high.”

The patient expressed satisfaction with the treatment outcomes, particularly regarding the restoration of a certain degree of daily functioning. She remarked, “Considering my progressively deteriorating condition a year ago, my family and I are extremely grateful that I can now independently perform daily activities.” The patient also emphasized the importance of regular follow-up appointments and treatment adherence, indicating her willingness to continue with the medical team’s treatment plan as prescribed.

## 4. Discussion

The pathogenesis of MDA5-associated RPILD involves complex interactions between environmental factors and dysregulated immune responses. MDA5, initially identified in DM patients with concurrent ILD by Sato et al in 2005,^[7]^ belongs to the retinoic acid-inducible gene I receptor family. These cytoplasmic proteins recognize viral double-stranded RNA, triggering antiviral defence mechanisms.^[8,9]^

In MDA5-associated RPILD, aberrant activation of this pathway leads to excessive interferon (IFN) production, creating a self-perpetuating cycle of inflammation. The abundant IFN further upregulates cytoplasmic MDA5 expression,^[10,11]^ potentially inducing autoimmune responses.^[12]^ Recent evidence demonstrates increased IFN-1 expression in alveolar epithelial cells of MDA5-positive DM patients.^[13]^ Transcriptomic analyses suggest a central role for the type I IFN pathway in MDA5 + DM pathogenesis, with RPILD patients exhibiting significantly elevated serum IFN-γ levels and strong IFN-γ-related immune responses in lung tissue and hilar lymph nodes.^[14]^

At the cellular level, while neutrophils, T lymphocytes, macrophages, and B lymphocytes all contribute to disease pathogenesis, B lymphocytes appear to play a pivotal role in MDA5-associated RPILD.^[15]^ Elevated levels of B-cell activating factor (BAFF) and a proliferation-inducing ligand (APRIL) have been observed in MDA5-positive patients. These cytokines regulate B-cell maturation, differentiation, and long-lived plasma cell antibody secretion.^[16]^

Clinical and experimental studies have elucidated the role of type I IFNs in inducing BAFF and APRIL production by dendritic cells and macrophages during viral infections.^[17]^ Lundell et al demonstrated that IFN-γ and IFN-α stimulation prompts BAFF secretion from basal cells in both basal and decidual membranes.^[18]^ Furthermore, Kato et al showed that double-stranded RNA induces IFN-β expression in airway epithelial cells, subsequently activating BAFF and contributing to autoimmune disease progression.^[19]^

T lymphocytes also play a crucial role in the pathogenesis of autoimmune diseases.^[20]^ Studies in rheumatoid arthritis have revealed that T cells can produce BAFF and APRIL, exerting functions similar to those of previously described cytokines.^[21]^ Yang et al demonstrated that activated T cells produce substantial amounts of BAFF, further promoting T cell proliferation and activation.^[22]^ Hu et al elucidated that BAFF influences T cell physiological activation through the BAFF receptor (BAFF-R)-mediated phosphoinositide 3-kinase-AKT signaling pathway,^[23]^ establishing a second self-perpetuating cycle of inflammation.

BAFF and APRIL exert their biological effects by binding to specific receptors. BAFF binds with decreasing affinity to BAFF-R, B-cell maturation antigen (BCMA), and TACI. APRIL binds sequentially to BCMA and TACI.^[24,25]^ These receptors are differentially expressed on B cell subsets, with BAFF-R predominant on naive, transitional, and memory B cells, TACI on marginal zone B cells and short-lived plasma cells, and BCMA on long-lived plasma cells.^[24]^

Targeting these ligand-receptor interactions presents a promising therapeutic strategy. Blocking BAFF and APRIL signaling could impede immature B cell development, inhibit memory cell survival, and reduce pathological cell circulation. Moreover, as TACI is expressed on both B and activated T lymphocytes,^[26,27]^ its blockade may concomitantly inhibit T cell activation, further attenuating the inflammatory cascade.

Telitacicept, a novel fully human TACI-Fc fusion protein, offers a mechanism to achieve this blockade. Developed using recombinant DNA technology, telitacicept links the extracellular domain of the B-cell surface receptor TACI with the Fc segment of immunoglobulin G1.^[28,29]^ Its dual-target effects on BAFF and APRIL modulate B lymphocyte maturation, differentiation, and autoantibody production. Additionally, by affecting BAFF-TACI interactions on activated T cells, telitacicept may inhibit T cell activation, achieving multi-stage suppression of both B and T lymphocyte functions.^[30]^

In comparison, rituximab, which targets CD20-positive B cells, cannot affect terminally differentiated plasmablasts and plasma cells due to their lack of CD20 expression.^[31]^ Consequently, antibody-producing plasma cells remain unaffected by rituximab treatment. Furthermore, rituximab’s specificity for B cells limits its impact on other cell types involved in MDA5-associated RPILD pathogenesis. Telitacicept’s broader mechanism of action may address these limitations, potentially offering a more comprehensive approach to disease management.

The case presented here, while encouraging, must be interpreted with caution. As a single case report, it provides limited clinical data and precludes definitive conclusions. The absence of certain biomarkers, such as KL-6, during the disease course limits our ability to fully assess disease activity and progression. Although lung CT scans indicated improvement in lung lesions, the extent of residual lung fibrosis remains uncertain. Moreover, the use of combination therapy, including steroids and cyclophosphamide alongside telitacicept, makes it challenging to isolate the specific contribution of telitacicept to the observed clinical improvement.

Despite these limitations, this case offers valuable insights into potential new treatment strategies for MDA5-associated RPILD. The positive outcome observed suggests that telitacicept, in combination with standard immunosuppressive therapy, may represent a promising avenue for managing this challenging condition. Future research directions should include larger-scale studies to evaluate the efficacy and safety of telitacicept in this patient population. Randomised controlled trials comparing telitacicept to standard of care or other biologics would be particularly informative. Additionally, long-term follow-up studies are necessary to assess the durability of treatment response and monitor for potential late-onset adverse effects.

Although no standardized treatment guidelines exist for anti-MDA5-associated RPILD, recent expert consensus statements recommend early combination immunosuppressive therapy as first-line treatment, typically comprising high-dose glucocorticoids with calcineurin inhibitors or cyclophosphamide.^[5,32]^ Our treatment approach largely aligned with these recommendations, employing pulse methylprednisolone and cyclophosphamide combination therapy. The addition of telitacicept represented a deviation from standard protocols, justified by the patient’s high-risk profile (advanced age, rapid progression) and evidence of significant B-lymphocyte activation on immunophenotyping. This approach is consistent with emerging recognition of anti-MDA5-positive DM as an “interferonopathy” requiring targeted therapy beyond conventional immunosuppression.^[33]^ Expert consensus acknowledges that rescue therapies may be individualized when standard approaches prove insufficient, supporting our rationale for incorporating novel therapeutic agents in this challenging case.

In conclusion, while further investigation is warranted, the successful treatment of this case with a regimen including telitacicept suggests a potentially promising approach for managing MDA5-associated RPILD, a condition historically associated with poor outcomes. The unique mechanism of action of telitacicept, targeting both B and T cell pathways, may offer a more comprehensive solution to the complex immunopathology of this disease.

## Author contributions

**Conceptualization:** Lang Zhang, Yuhan Luo, Xingqin Shao, Rongrong Hu, Yan Zha.

**Data curation:** Lang Zhang, Yuhan Luo, Rongrong Hu, Yan Zha.

**Formal analysis:** Lang Zhang, Xingqin Shao.

**Investigation:** Lang Zhang, Yuhan Luo.

**Methodology:** Lang Zhang.

**Project administration:** Lang Zhang.

**Resources:** Lang Zhang, Yuhan Luo, Jing Yuan.

**Software:** Lang Zhang, Yuhan Luo, Jing Yuan.

**Supervision:** Lang Zhang, Yuhan Luo.

**Validation:** Lang Zhang.

**Visualization:** Lang Zhang, Rongrong Hu.

**Writing – original draft:** Lang Zhang, Xingqin Shao, Yan Zha, Xin Lin.

**Writing – review & editing:** Xin Lin.

## Supplementary Material

SUPPLEMENTARY MATERIAL
