# Supplementary material for: Telitacicept combined with conventional therapy successfully treated MDA5-associated RPILD: A case report
Source: Medicine (Baltimore). 2025 Jul 11;104(28):e43293. doi: 10.1097/MD.0000000000043293 (PMC12262947; doi:10.1097/MD.0000000000043293)
Supplement: SUPPLEMENTARY MATERIAL [file medi-104-e43293-s001.docx]

**Supplementary Figure S1: Baseline chest CT examination**


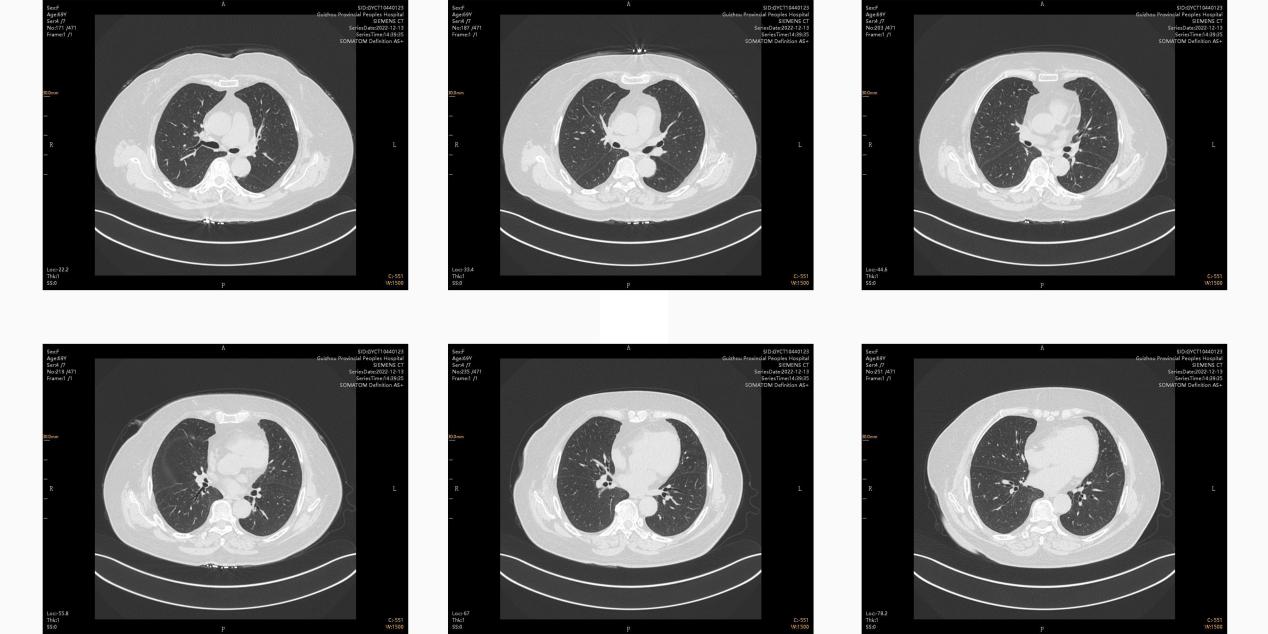


(d)

(f)

(e)

(c)

(b)

(a)

****Caption:** Baseline chest CT scan performed on December 13, 2022 (approximately 10 months prior to acute presentation). Images show bilateral lung nodular changes with limited ground-glass opacities. The radiological report documented: bilateral upper lobe consolidations (left upper lobe approximately 3×3mm, right upper lobe approximately 5×4mm), bilateral lower lobe subpleural nodular high-density shadows measuring approximately 7mm, and scattered bilateral nodular shadows. Multiple lymph nodes were observed in bilateral hilar regions. This baseline imaging demonstrates the previously stable, limited disease extent, highlighting the rapid progressive nature of MDA5-associated interstitial lung disease during the acute presentation.**
